# Comparative adipokine profile in middle-aged master athletes, untrained adults, and young individuals

**DOI:** 10.1007/s00421-026-06425-0

**Published:** 2026-09-12

**Authors:** Patrício Lopes de Araújo Leite, Larissa Alves Maciel, Márcia Cristiane Araújo, Isabella Ferreira Heringer Pena, João Vitor Sena de Carvalho, Walliyson Sousa de Lima, Marcus Vinicius Alves Pereira, Ester Rodrigues De Moura, Kadson Kauã Monteiro Dias, Pietra Campbell Simões, Elbert Wander Cantão, Carmen Silvia Grubert Campbell, Isabela Almeida Ramos, Rodrigo Vanersson Passos Neves, Thiago dos Santos Rosa, Herbert Gustavo Simões

**Affiliations:** 1 Graduate Program in Physical Education, Catholic University of Brasilia, Taguatinga, DF Brazil; 2 Genomic Sciences and Biotechnology Program, Catholic University of Brasilia, Taguatinga, DF Brazil; 3 Human Movement Sciences Program, State University of Northern Paraná, Bandeirantes, PR Brazil

**Keywords:** Metabolism, Aging, Athletics, Adipose tissue

## Abstract

**Background:**

Adipose tissue is an active endocrine organ involved in metabolic regulation and systemic inflammation, processes that are altered during aging. Nevertheless, the adipokine profile associated with lifelong physical training in master.

athletes remains incompletely characterized.

**Aim:**

This study investigated circulating concentrations of adiponectin, leptin, resistin, insulin, and the leptin-to-adiponectin and adiponectin-to-resistin ratios in four male groups: endurance master athletes (ME), master sprinters (MS), untrained middle-aged adults (UMA), and untrained young adults (YU).

**Methods:**

ME (n = 17, 50.5 ± 9.6 years), MS (n = 30, 50.3 ± 6.2 years), UMA (n = 16, 48.5 ± 7.5 years), and YU (n = 23, 23.7 ± 4.0 years) underwent anamnesis, anthropometric assessments, and serum concentrations of adiponectin, leptin, resistin, and insulin using commercial ELISA kits.

**Results and discussion:**

Significant group differences were observed for all variables (p < 0.001). UMA exhibited higher concentrations of leptin, insulin, and resistin, together with an elevated leptin-to-adiponectin ratio and a reduced adiponectin-to-resistin ratio compared with the other groups (p < 0.05). MS also presented higher leptin and insulin levels and an increased leptin-to-adiponectin ratio compared.

with YU (p < 0.05), as well as a lower adiponectin-to-resistin ratio (p < 0.05). Overall, effect sizes were large across outcomes, and master athletes displayed a more favorable adipokine profile than untrained middle-aged adults, suggesting potential metabolic adaptations associated with lifelong training.

**Conclusions:**

Middle-aged master athletes exhibited a more favorable adipokine profile than untrained middle-aged adults, with values approaching those observed in young individuals.

## Introduction

Aging is associated with alterations in adipokine regulation, often characterized by lower adiponectin and increased leptin, resistin, and insulin concentrations. These alterations, further exacerbated by sedentary behavior, contribute to a progressively unfavorable metabolic profile and to a chronic low-grade inflammatory state known as inflammaging (Alisson et al., 2012; Goyal and Rakhra 2024; Dugan et al. 2023). In this context, adipose tissue functions as an active endocrine organ, secreting bioactive molecules that regulate metabolic homeostasis and inflammatory signaling and are increasingly recognized as biomarkers of biological aging (Arai et al. 2011).

Among these molecules, adiponectin exerts anti-inflammatory and insulin-sensitizing effects and contributes to metabolic regulation and energy homeostasis (Fang and Judd 2018). In contrast, leptin and resistin are more closely associated with adiposity, inflammation, and metabolic dysfunction ( Münzberg et al. 2024; Acquarone et al. 2019; Taouis, 2021). Age-related insulin resistance further contributes to this adverse metabolic environment (Kolb et al. 2023; Norton et al. 2022). Because these adipokines operate within an interconnected regulatory network, the leptin-to-adiponectin and adiponectin-to-resistin ratios have been proposed as integrated indicators of metabolic and inflammatory balance (Pangaribuan et al. 2011; Chou et al. 2014), potentially providing complementaryinformation beyond isolated adipokine concentrations.

Lifelong physical training has been associated with improved body composition, reduced systemic inflammation, and enhanced metabolic regulation during aging (Barbosa et al. 2021; Aguiar et al. 2020; Minuzzi et al. 2024; Rosa et al. 2020; Sousa et al. 2019; Leite et al. 2024; Leite et al. 2023; Aguiar et al. 2021a, b). Master athletes, defined as individuals over 35 years of age who have engaged in structured training and competitive sport over decades, represent a unique human model for investigating mechanisms associated with healthy aging.

However, evidence specifically examining adipokine profiles in master athletes remains limited. To the best of our knowledge, no previous study has simultaneously evaluated adiponectin, leptin, resistin, and their integrative ratios in master athletes, particularly across sprint and endurance disciplines. This knowledge gap limits a more comprehensive understanding of how lifelong training and different training backgrounds may influence adipokine regulation.

Therefore, the present study compared circulating concentrations of adiponectin, leptin, resistin, insulin, and their respective ratios in endurance master athletes, master sprinters, untrained middle-aged adults, and young controls. We hypothesized that master athletes would exhibit a more favorable adipokine profile compared with untrained middle-aged adults.

## Methods

This cross-sectional observational study was approved by the Research Ethics Committee for Human Studies at the Catholic University of Brasília (protocol number 3,779,535) and conducted in accordance with the principles of the Declaration of Helsinki and its later amendments. All participants volunteered and provided written informed consent after receiving a full explanation of the study procedures.

### Participants

The sample included four male groups: master sprinters (MS), master endurance runners (ME), untrained young adults (YU), and untrained middle-aged adults (UMA). Master athletes were eligible if they were men between 40 and 75 years of age, non-smokers, free from metabolic, inflammatory, cardiovascular, or neoplastic diseases, and not engaged in the regular use of medications, including hormonal therapies.

Master athletes were required to be actively engaged in systematic training in their primary competitive discipline and to participate in master athletics competitions during the year of data collection, including regional, national, or international events. No minimum number of training years was established as an inclusion criterion, although most participants reported a substantial training history. Athletes were classified according to their primary competitive event as master sprinters (≤400 m) or master endurance athletes (≥800 m). Training specificity was defined according to the discipline in which participants primarily trained and competed during the period of data collection.

Previous participation in other sports was not an exclusion criterion, and no predefined proportion of weekly training volume was used to determine modality classification.

Participants were also required to report no period of physical inactivity longer than six months during their training history. Untrained participants were included if they met the same general clinical and lifestyle criteria applied to the athlete groups and reported neither a history of regular sports participation, including competitive participation, lasting more than one year at any point in their lives, nor any regular sports participation during the two years preceding data collection.

Individuals from any group were excluded if they had experienced a recent infection within the past 30 days, reported the use of anti-inflammatory agents or corticosteroids, or had any diagnosed metabolic or cardiovascular condition. Master athletes were recruited during national, regional, or world master athletics competitions and through personal referrals, whereas untrained individuals were recruited via community-based convenience sampling.

### General procedures

Data collection occurred in the morning (7-9 a.m.), either during master athletics competitions or in the laboratory, depending on participant availability. All volunteers arrived after an overnight fast lasting between eight and twelve hours, abstained from vigorous physical activity for at least twelve hours, and avoided alcohol and caffeine for twenty-four hours.

Upon arrival, participants completed a structured anamnesis addressing medical history, physical activity habits, and training routines, followed by anthropometric assessments and venous blood sampling.

Among the master sprinters, 19 were assessed during competition periods at national or international events, including the Brazilian, South American, and World Championships, whereas 11 were assessed outside competition periods in a local laboratory. All 17 endurance master athletes were assessed outside competition periods in the local laboratory. To examine whether the assessment setting and competition period influenced the outcomes, the master sprinters were subdivided according to whether they were assessed during or outside competition. Adipokines, insulin, and the calculated adipokine ratios and indices were compared between these two master sprinters subgroups and between each subgroup and the endurance athletes.

### Anthropometry and body composition

Body mass and height were assessed using a digital scale (Filizola^®^, Brazil) and a fixed stadiometer (Sanny^®^, Brazil). Body mass index was calculated subsequently. Subcutaneous fat was evaluated at seven anatomical sites using a Lange^®^ skinfold caliper (Cambridge Scientific Instruments, USA), with all measurements collected by the same trained evaluator. Each site was measured three times, and the mean value was used for analysis.

Relative body fat was estimated using the Jackson and Pollock equations (1978), and body density was converted to percent body fat using the Siri formula (Siri 1961).Fat-free mass was then derived from these estimates.

### Blood collection and biochemical analyses

Approximately eight milliliters of venous blood were collected using Vacutainer® tubes. Serum was separated by centrifugation (1500g, 15 min). Serum concentrations of adiponectin, leptin, resistin, and insulin were determined using commercially available ELISA kits validated for human samples. All samples were analyzed in duplicate within the same analytical batch to minimize variation. The detection limits for the assays were 132 pg/mL and 1.1 pmol/L, respectively. Intra- and inter-assay coefficients of variation were below 14%. The leptin-to-adiponectin and adiponectin-to-resistin ratios were calculated for each participant.

### Statistical analysis

Data normality was assessed for all continuous variables, including demographic, anthropometric, metabolic, and adipokine-related outcomes, separately within each study group using the Shapiro–Wilk test. Because several variables showed significant deviations from normalitin at least one group, between-group differences were analyzed using the Kruskal–Wallis test. When a significant overall group effect was detected, pairwise comparisons were performed using Dunn’s post hoc test with Bonferroni adjustment for multiple comparisons. Effect sizes for the Kruskal–Wallis tests were calculated using epsilon squared (ε^2^) and interpreted as approximately 0.01, small; 0.06, moderate; and 0.14, large. Demographic and anthropometric characteristics are presented as mean ± standard deviation, whereas metabolic and adipokine-related variables are presented as median and interquartile range. All inferential between-group comparisons were performed using rank-based nonparametric tests. As an additional sensitivity analysis, master sprinters were subdivided according to whether they were assessed during competition or outside competition in a local laboratory. Adipokines, insulin, and the calculated adipokine ratios and indices were compared among master sprinters assessed during competition, master sprinters assessed outside competition, and endurance athletes using the same nonparametric procedures described above. All statistical tests were two-tailed, and statistical significance was set at p < 0.05. Analyses were performed using GraphPad Prism, version 8.0, and IBM SPSS Statistics, version 30.

## Results

The main characteristics of the study groups are presented in Table 1. Significant differences were observed between groups for age, body mass, height, BMI, and body fat percentage. As expected, age was significantly lower in YU than in UMA, MS, and ME (p < 0.05). Body mass was higher in UMA than in MS and ME (p < 0.05). Height differed between groups, with ME presenting lower values compared with UMA (p < 0.05).

BMI was significantly higher in UMA compared with YU, MS, and ME (p <0.05). Similarly, body fat percentage was greater in UMA compared with the other groups (p < 0.05). No significant differences were observed between MS and ME for BMI or body fat percentage (Table 1).

**Fig. 1:**
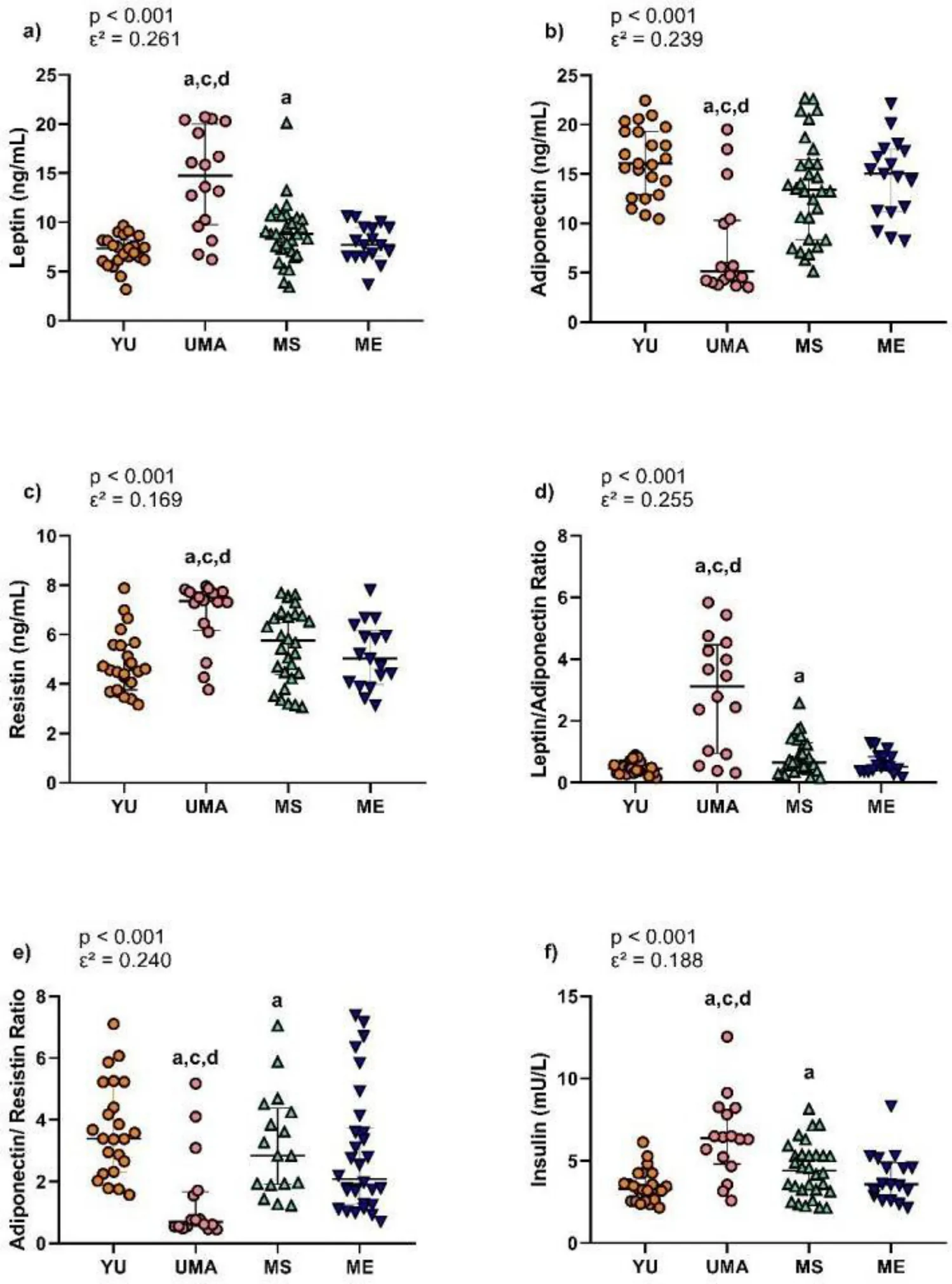
Circulating concentrations of **a** leptin, **b** adiponectin, **c** resistin, **d** leptin-to-adiponectin ratio, **e** adiponectin-to-resistin ratio, and **f** insulin in young untrained adults (YU), untrained middle-aged adults (UMA), master sprinters (MS), and master endurance athletes (ME). Individual data points are presented with median and interquartile range. The global *p* value from the Kruskal–Wallis test and the corresponding epsilon-squared effect size (ε^2^) are shown in each panel. Superscript letters indicate significant pairwise differences from the corresponding group: **a** YU, **b** UMA, **c** MS, and **d** ME, according to Dunn’s post hoc test with Bonferroni adjustment (*p* <0.05)

Leptin concentrations differed significantly among groups (H(3) = 24.39, p <0.001, ε^2^ = 0.261), with UMA exhibiting higher leptin concentration compared with YU, MS, and ME (p < 0.05), while MS also showed higher values than YU (p < 0.05) (Figure1a).

Adiponectin concentrations also differed between groups (H(3) = 22.56, p <0.001, ε^2^ = 0.239), with UMA showing lower adiponectin concentration compared with YU, MS, and ME (p < 0.05) (Figure 1b).

Resistin concentrations were significantly different across groups (H(3) = 16.84, p < 0.001, ε^2^ = 0.169), with UMA showing higher resistin concentration than YU, MS, and ME (p < 0.05) (Figure 1c).

The leptin-to-adiponectin ratio differed significantly between groups (H(3) =23.89, p < 0.001, ε^2^ = 0.255), with UMA displaying a higher ratio compared with YU, MS, and ME (p < 0.05), and MS also differed from YU (p < 0.05) (Figure 1d).

The adiponectin-to-resistin ratio showed significant group differences (H(3) =22.70, p < 0.001, ε^2^ = 0.240), with UMA exhibiting lower values compared with YU, MS, and ME (p < 0.05), and MS differed from YU (p < 0.05) (Figure 1e).

Insulin concentrations differed significantly among groups (H(3) = 18.42, p <0.001, ε^2^ = 0.188), with UMA presenting higher insulin levels compared with YU, MS, and ME (p < 0.05), and MS also differed from YU (p < 0.05) (Figure 1f).

### Overall, effect sizes were large across outcomes

As an additional sensitivity analysis, the master sprinters were subdivided according to the assessment condition. Nineteen master sprinters (63.3%) were assessed during competition periods, whereas 11 (36.7%) were assessed outside competition in a local laboratory. All 17 endurance master athletes were assessed outside competition in the local laboratory. No significant differences were observed between master sprinters assessed during and outside competition, or between either master sprinters subgroup and the endurance group, for adiponectin, leptin, resistin, insulin, or any of the calculated adipokine ratios and indices (all p > 0.05).

## Discussion

This study investigated adipokine profiles and integrative adipokine ratios in master athletes from different training modalities. The main finding was that master athletes exhibited a more favorable adipokine and insulin profile compared with untrained middle-aged adults, characterized by lower circulating concentrations of leptin, resistin, and insulin, as well as more favorable leptin-to-adiponectin and adiponectin-to-resistin ratios. For several variables, values in master athletes approached those observed in young adults, suggesting that lifelong training may attenuate age-related metabolic alterations.

The higher leptin concentrations observed in the UMA group are consistent with the greater body fat percentage identified in this group. Previous studies involving master athletes have consistently reported lower adiposity and more favorable body composition compared with age-matched untrained individuals, reflecting the long-term influence of regular training on body fat regulation (Simões et al. 2017; Aguiar et al. 2021a, b). Because leptin concentrations are closely related to adipose tissue mass, these findings likely reflect differences in body composition between groups. In addition, chronic hyperleptinemia has been associated with metabolic dysregulation and low-grade inflammation, further supporting leptin as a link between adiposity and metabolic health (Klein et al. 1996).

In parallel, hyperinsulinemia reflects peripheral insulin resistance and is frequently associated with increased adiposity and chronic low-grade metabolic inflammation (Kumar et al. 2018). The lower insulin concentrations observed in master athletes are therefore consistent with improved metabolic regulation. Consistent with this perspective, previous evidence has reported lower circulating insulin concentrations in master athletes compared with non-athletic individuals, supporting the notion that long-term training is associated with improved insulin sensitivity and metabolic regulation (Aguiar et al. 2022).

Resistin has been associated with inflammatory signaling and metabolic dysfunction. In humans, it is primarily secreted by immune cells and has been linked to chronic low-grade inflammation (Taouis 2021). The lower resistin concentrations observed in master athletes may therefore reflect reduced inflammatory activation associated with long-term training.

Adiponectin plays an important role in metabolic regulation and has been widely associated with improved insulin sensitivity and anti-inflammatory effects (Fang and Judd 2018). Although the so-called adiponectin paradox has been proposed in certain clinical conditions (Baker et al. 2019; Menzaghi and Trischitta 2018), recent evidence suggests that higher adiponectin concentrations may still reflect a more favorable metabolic phenotype in healthy populations (Kim et al. 2025). In this context, the preservation of adiponectin concentrations observed in master athletes may reflect a metabolic state characterized by greater metabolic flexibility and reduced inflammatory stress.

Nevertheless, this interpretation should be made cautiously because adiponectin and leptin may increase in the setting of impaired kidney function due to reduced renal clearance (Korczynska et al. 2021; Heidari et al. 2015). Since renal biomarkers such as serum creatinine, estimated glomerular filtration rate, and albuminuria were not assessed, a potential influence of kidney function on circulating adipokine concentrations cannot be completely excluded. However, previous evidence in comparable cohorts of master athletes has reported preserved estimated kidney function in trained individuals compared with untrained middle-aged adults, making it less likely that the adipokine profile observed in the athletes reflects the renal adiponectin paradox (Rosa et al. 2021).

Moreover, the overall pattern observed here, characterized by lower leptin, resistin, and insulin concentrations together with more favorable adipokine ratios and lower adiposity in athletes, is more consistent with an improved metabolic phenotype than with adipokine disturbances typically described in advanced chronic kidney disease.

The analysis of adipokine ratios provides an integrated perspective on the balance between metabolic regulation and inflammatory activity. In particular, the leptin-to-adiponectin ratio has been widely used as an indicator of metabolic dysfunction, as elevated values are associated with reduced insulin sensitivity and increased type 2 diabetes risk (Thorand et al. 2010; Oda et al. 2008). In contrast, the adiponectin-to-resistin ratio may reflect the balance between anti-inflammatory and pro-inflammatory adipokine signaling (Fargnoli et al. 2010). In the present study, the more favorable ratios observed in athletes suggest an endocrine-metabolic profile characterized by lower systemic inflammation and improved metabolic regulation.

The absence of differences between endurance and master sprinters suggests that cumulative exposure to long-term training, regardless of the predominance of aerobic or anaerobic pathways, may be sufficient to promote convergent adaptations in immunometabolic regulation. Lifelong engagement in systematic training may therefore contribute to the maintenance of metabolic homeostasis and attenuation of low-grade inflammation during aging (Aguiar et al. 2020; Barbosa et al. 2021). Nevertheless, specific characteristics of each training modality, such as differences in training volume and intensity, may induce distinct physiological adaptations that were not detected in the present study. Thus, further research is warranted to clarify potential modality-specific effects on adipokine signaling and metabolic regulation.

Some limitations should be considered when interpreting these findings. First, the cross-sectional design precludes causal inference regarding the long-term effects of training on adipokine profiles. Second, body composition was estimated using skinfold thickness rather than a gold-standard technique such as dual-energy X-ray absorptiometry (DXA). Habitual dietary intake was also not controlled, although it may influence circulating adipokine concentrations. In addition, the study included only male participants, which limits the generalizability of the findings.

A further important limitation is the inability to fully disentangle the effects of age, training status, and adiposity. The UMA group had higher body mass, BMI, and body fat percentage than the other groups, which may have contributed to its less favorable metabolic and adipokine profile. Although stratification by weight status could have helped clarify this relationship, the small sample size and unequal distribution of participants across BMI categories would have produced small and unbalanced subgroups, limiting statistical power and the robustness of the estimates. Therefore, the observed differences cannot be attributed exclusively to aging or long-term training.

Future studies should include larger samples and groups matched or adequately stratified according to adiposity. Despite these limitations, this study presents important strengths. To the best of our knowledge, this is one of the first investigations to examine adiponectin, leptin, resistin, and insulin in an integrated manner in master athletes. The inclusion of highly trained athletes with long-term competitive participation provides a unique model to investigate physiological adaptations associated with lifelong training and healthy aging.

## Conclusion

Master athletes exhibited lower circulating concentrations of leptin, resistin, and insulin and more favorable adipokine ratios compared with untrained middle-aged adults. These findings indicate distinct adipokine profiles among trained and untrained groups and support the relevance of master athletes as a model for studying biological characteristics associated with long-term training during aging.

## Data Availability

The datasets collected and analysed during the current study are not publicly available due to ethical andprivacy considerations related to the protection of participant confidentiality. De-identified data may be made available by the corresponding author uponreasonable request, subject to applicable ethical requirements.
